# Supplementary figures and images for: Development and validation of a novel risk score to predict 5-year mortality in patients with acute myocardial infarction in China: a retrospective study
Source: PeerJ. 2022 Jan 4;10:e12652. doi: 10.7717/peerj.12652 (PMC8740514; doi:10.7717/peerj.12652)

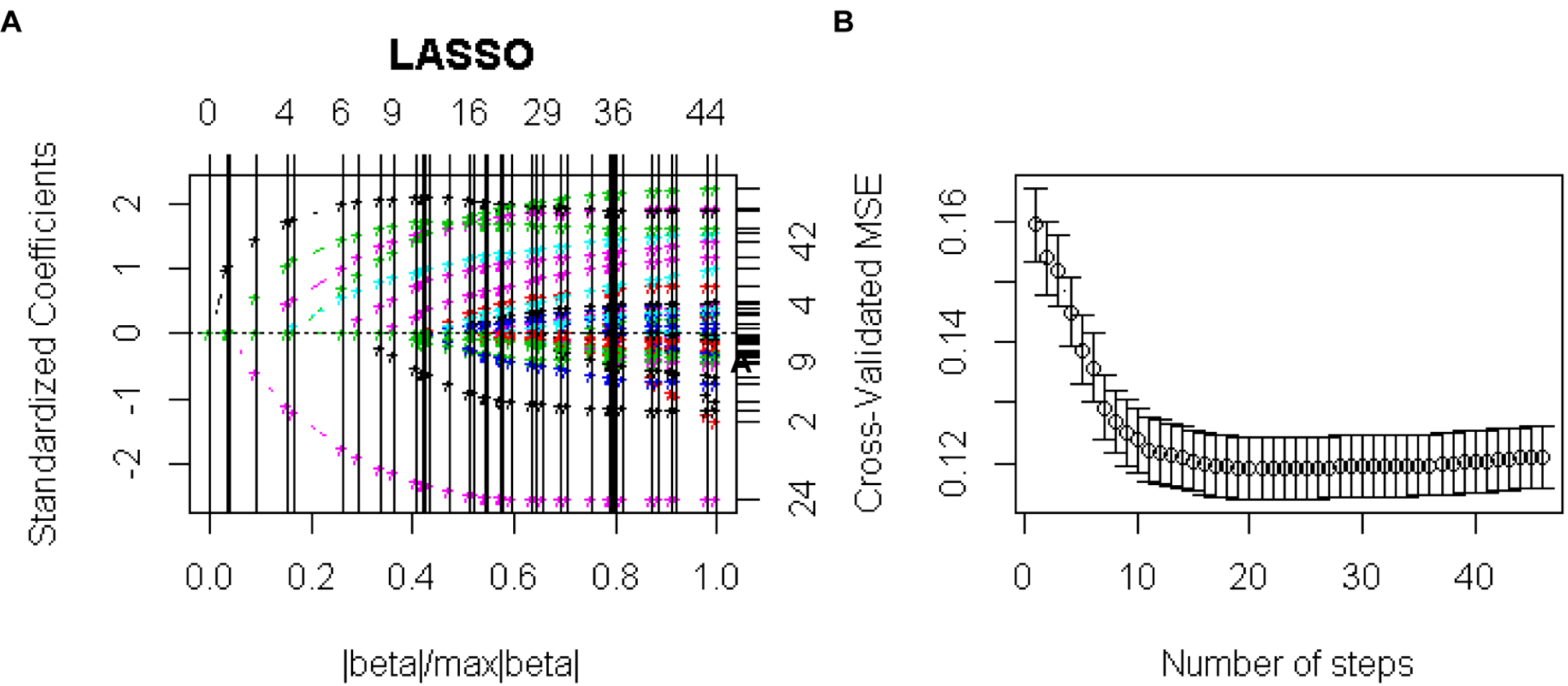

Supplement: Supplemental Information 1 — (A) LASSO Coefficient Profiles of the 45 Baseline Variables. (B) LASSO Regularization Level was Chosen by 10-fold Cross-Validation Using the one Standard Error Rule. Abbreviations: LASSO, least absolute shrinkage and selection operator. [file peerj-10-12652-s001.png]

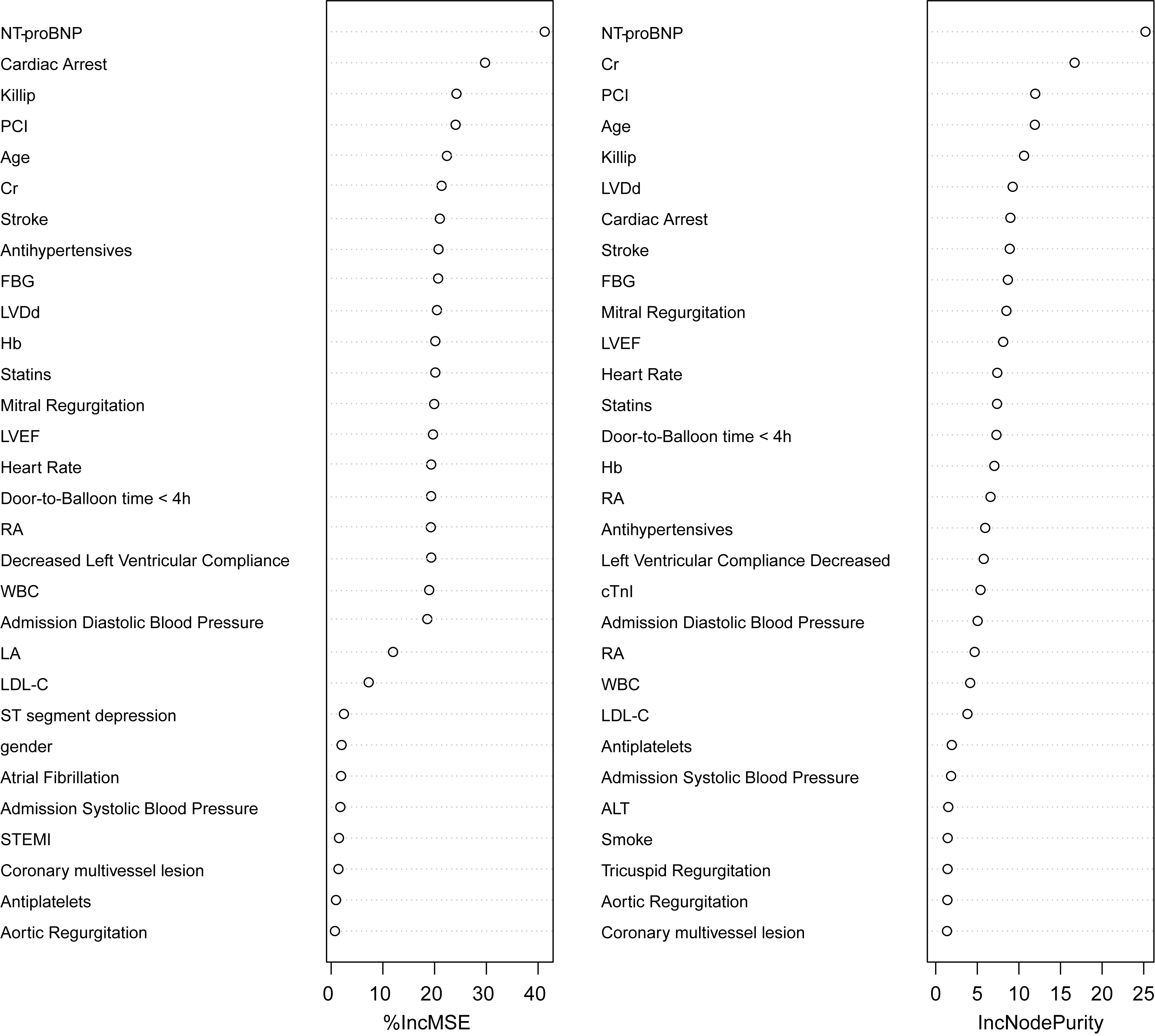

Supplement: Supplemental Information 2 — Abbreviations: MSE, mean squared error; STEMI, ST segment elevation myocardial infarction; Door-to-Balloon time, Time from hospital arrival to first balloon inflation; WBC, white blood cell; Hb, hemoglobin; ALT, alanine transaminase; Cr, creatinine; FBG, fast blood glucose; LDL-C, low-density lipoprotein cholesterol; NT-proBNP, N-terminal pro-brain natriuretic peptide; cTnI, cardiac Troponin I; LVEF, left ventricular ejection fraction; LA, left atrial; LVDd, left ventricular end-diastolic diameter; RA, right atrial; Antiplatelets, aspirin, clopidogrel, ticagrelor; Antihypertensives, angiotensin-converting enzyme inhibitor, angiotensin receptor blocker, calcium-channel blocker, β-receptor blocker; PCI, percutaneous transluminal coronary intervention. [file peerj-10-12652-s002.png]

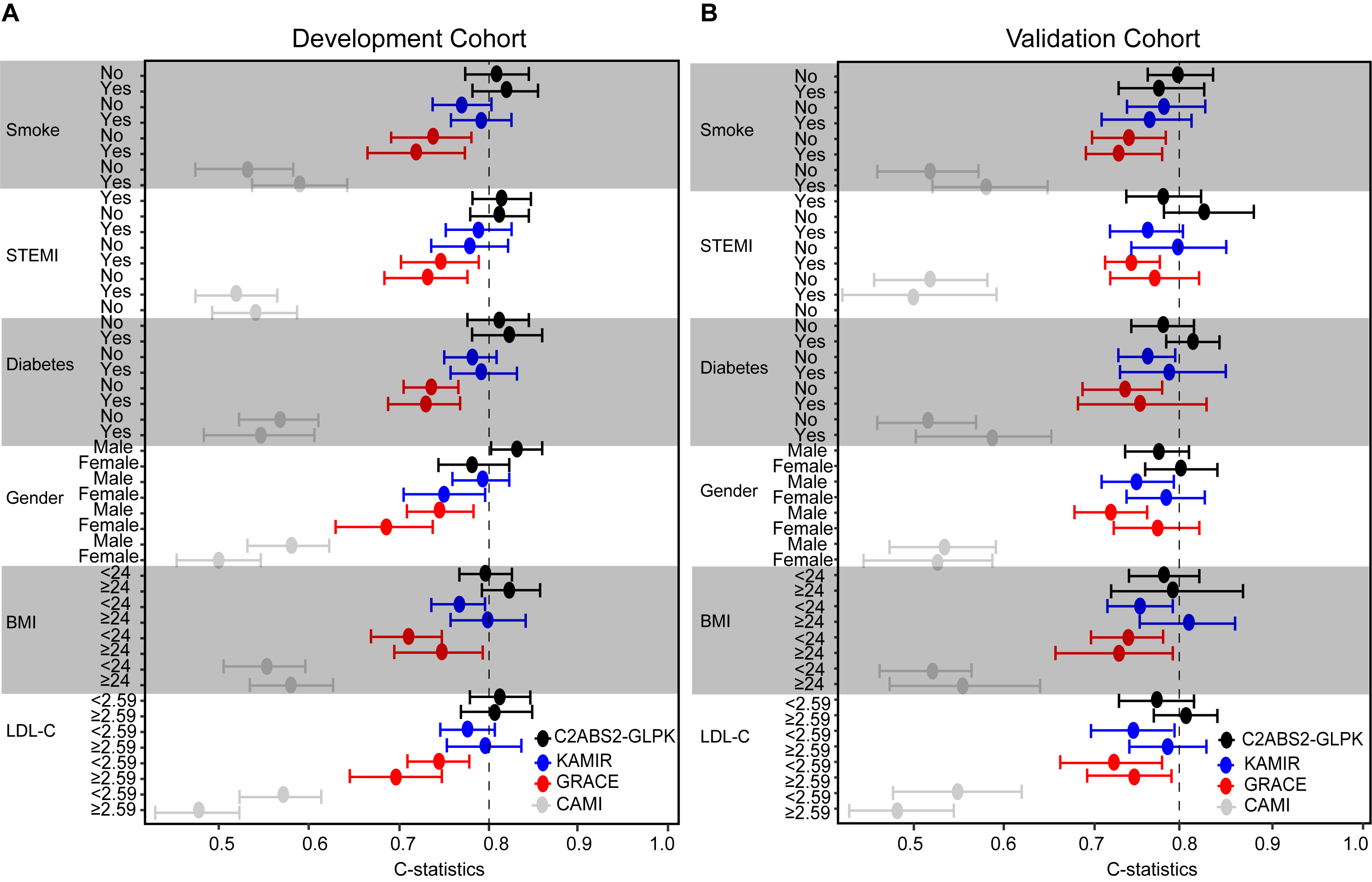

Supplement: Supplemental Information 3 — (A) Subpopulation C-statistics Analysis for C2ABS2-GLPK, GRACE, KAMIR and CAMI Scores in the Development Cohort. (B) Subpopulation C-statistics Analysis for C2ABS2-GLPK, GRACE, KAMIR and CAMI Scores in the External Validation Cohort. Notes: The Dashed Vertical Line Represents the C-statistics in the Total Population (0.80). Horizontal Lines Represent 95% CI. Abbreviations: GRACE, Global Registry of Acute Coronary Events; KAMIR, Korea Acute Myocardial Infarction Registry; CAMI, China Acute Myocardial Infarction. [file peerj-10-12652-s003.png]
