# Supplementary material for: Development and validation of a novel risk score to predict 5-year mortality in patients with acute myocardial infarction in China: a retrospective study
Source: PeerJ. 2022 Jan 4;10:e12652. doi: 10.7717/peerj.12652 (PMC8740514; doi:10.7717/peerj.12652)
Supplement: Supplemental Information 5 — Abbreviations: BMI, body mass index; STEMI, ST segment elevation myocardial infarction; MI, myocardial infarction; Door-to-Balloon time, Time from hospital arrival to first balloon inflation;SBP, systolic blood pressure; DBP, diastolic blood pressure; WBC, white blood cell; Hb, Hemoglobin; PLT, platelet; ALT, alanine transaminase; FBG, fast blood glucose; LDL-C, low-density lipoprotein cholesterol; NT-proBNP, N-terminal pro-brain natriuretic peptide; CK-MB, creatine kinase isoenzyme; cTnI, cardiac troponin I; LVEF, left ventricular ejection fraction; LA, left atrial; LVDd, left ventricular end-diastolic diameter; RA, right atrial; RV, right ventricular; LV, left ventricular; Antiplatelets, aspirin, clopidogrel, ticagrelor; Antihypertensives, angiotensin-converting enzyme inhibitor, angiotensin receptor blocker, calcium-channel blocker, β-receptor blocker; PCI, percutaneous transluminal coronary intervention; CABG, coronary artery bypass grafting. [file peerj-10-12652-s005.doc]

**Table S2. Baseline Variables Included for Variable Selection Process.**

| **Variables** | **Evidence and/or models** | **Inclusion/ exclusion** |
| --- | --- | --- |
| Age,y | Grace (*Fox et al., 2010*), KAMIR(*Kim et al., 2011*), TIMI (*Morrow et al., 2000*) | Included |
| Male, n (%) | CAMI (*Xu* *et al., 2016*) | Included |
| BMI, kg/m^2^ | CAMI (*Xu* *et al., 2016*) | Included |
| Current smoking, n (%) | CAMI (*Xu* *et al., 2016*) | Included |
| ST segment depression, n (%) | Grace (*Fox et al., 2010*) | Included |
| STEMI, n (%) | CAMI (*Xu* *et al., 2016*) | Included |
| Acute Anterior MI, n (%) | CAMI (*Xu* *et al., 2016*) | Included |
| Left main coronary lesion, n (%) | Genisini (*Peterson et al., 2008*) | Included |
| Coronary multivessel lesion, n (%) | Genisini (*Peterson et al., 2008*) | Included |
| Door-to-Balloon time > 4h (yes vs no) | TIMI (*Morrow et al., 2000*) | Included |
| Cardiac Arrest (yes vs no) | Grace (*Fox et al., 2010*), CAMI (*Xu* *et al., 2016*) | Included |
| Killip, classifications | Grace (*Fox et al., 2010*), TIMI (*Morrow et al., 2000*) | Included |
| Heart Rate, beats/min | Grace (*Fox et al., 2010*), TIMI (*Morrow et al., 2000*), CAMI (*Xu* *et al., 2016*) | Included |
| SBP, mmHg | Grace (*Fox et al., 2010*), TIMI (*Morrow et al., 2000*) | Included |
| DBP, mmHg | Abnormal blood tests associated with poor outcomes of AMI (*Park et al., 2017*) | Included |
| WBC, *10^9^/L | CAMI (*Xu et al., 2016*) | Included |
| Hb, g/L | CHA2DS2-VASc-CF Score (*Kundi et al., 2018*) | Included |
| PLT, *10^9^/L | Abnormal blood tests associated with poor outcomes of AMI (*Avramakis et al., 2007*) | Included |
| ALT, U/L | Abnormal blood tests associated with poor outcomes of AMI (*Lazzeri et al., 2010*) | Included |
| Creatinine, μmol/L | Grace (*Fox et al., 2010*), KAMIR (*Kim et al., 2011*) | Included |
| FBG, mmol/L | KAMIR (*Kim et al., 2011*) | Included |
| LDL-C, mmol/L | CHA2DS2-VASc-CF Score (*Kundi et al., 2018*) | Included |
| NT-proBNP, pg/ml | Abnormal blood tests associated with poor outcomes of AMI (*Takahashi et al., 2020*) | Included |
| CK-MB, IU/L | Grace (*Fox et al., 2010*) | Excluded–too many values missing from derivation dataset |
| cTnI, μg/L | Grace (*Fox et al., 2010*) | Included |
| Abnormal thyroid function (yes vs no) | Abnormal hormone tests associated with poor outcomes of AMI (*She et al., 2018*) | Excluded–too many values missing  from derivation dataset |
| Prior history of angina (yes vs no) | TIMI (*Morrow et al., 2000*) | Excluded–too many values missing  from derivation dataset |
| Prior history of acute myocardial infarction (yes vs no) | TIMI (*Morrow et al., 2000*), CAMI (*Xu et al., 2016*) | Included |
| Prior history of congestive heart failure (yes vs no) | The GRACE 6-month postdischarge prediction model (*Eagle et al., 2004*) | Excluded–too many values missing  from derivation dataset |
| Prior history of PCI (yes vs no) | CAMI (*Xu* *et al., 2016*) | Included |
| History of Hypertension (yes vs no) | CHA2DS2-VASc-CF Score (*Kundi et al., 2018*) | Included |
| History of Diabetes (yes vs no) | TIMI (*Morrow DA et al., 2000*), CHA2DS2-VASc-CF Score (*Kundi et al., 2018*) | Included |
| History of Stroke (yes vs no) | Comorbidity predicts clinical outcomes in AMI (*Brammås et al., 2013*) | Included |
| History of Atrial Fibrillation (yes vs no) | Abnormal electrocardiography tests associated with poor outcomes of AMI (*Lee et al., 2020*) | Included |
| LVEF, % | KAMIR (*Kim et al., 2011*) | Included |
| LA, mm | Abnormal echocardiography tests associated with poor outcomes of AMI (*Solomon et al., 2005*) | Included |
| LVDd, mm | Abnormal echocardiography tests associated with poor outcomes of AMI (*Solomon et al., 2005*) | Included |
| RA, mm | Abnormal echocardiography tests associated with poor outcomes of AMI (*Solomon et al., 2005*) | Included |
| RV, mm | Abnormal echocardiography tests associated with poor outcomes of AMI (*Solomon et al., 2005*) | Included |
| Aortic Regurgitation, n (%) | Abnormal echocardiography tests associated with poor outcomes of AMI (*Solomon et al., 2005*) | Included |
| Mitral Regurgitation, n (%) | Abnormal echocardiography tests associated with poor outcomes of AMI (*Solomon et al., 2005*) | Included |
| Tricuspid Regurgitation, n (%) | Abnormal echocardiography tests associated with poor outcomes of AMI (*Solomon et al., 2005*) | Included |
| Pulmonary Regurgitation, n (%) | Abnormal echocardiography tests associated with poor outcomes of AMI (*Solomon et al., 2005*) | Included |
| Decreased LV Compliance, n (%) | Abnormal echocardiography tests associated with poor outcomes of AMI (*Solomon et al., 2005*) | Included |
| Antiplatelet Use (yes vs no) | Abnormal treatments associated with poor outcomes of AMI (*Tomaniak et al., 2019*) | Included |
| Statins Use (yes vs no) | Abnormal treatments associated with poor outcomes of AMI (*Kim et al., 2019*) | Included |
| Antihypertensive Use (yes vs no) | Abnormal treatments associated with poor outcomes of AMI (*Barons et al., 2015*) | Included |
| PCI | Grace (*Fox et al., 2010*), KAMIR (*Kim et al., 2011)*, TIMI *(Morrow et al., 2000*) | Included |
| CABG | ARTS (*Serruys PW et al., 2005*) | Included |

**Abbreviations:** BMI: body mass index; STEMI: ST segment elevation myocardial infarction; MI: myocardial infarction; Door-to-Balloon time: Time from hospital arrival to first balloon inflation;SBP: systolic blood pressure; DBP: diastolic blood pressure; WBC: white blood cell; Hb: Hemoglobin; PLT: platelet; ALT: alanine transaminase; FBG: fast blood glucose; LDL-C: low-density lipoprotein cholesterol; NT-proBNP: N-terminal pro-brain natriuretic peptide; CK-MB: creatine kinase isoenzyme; cTnI: cardiac troponin I; LVEF: left ventricular ejection fraction; LA: left atrial; LVDd: left ventricular end-diastolic diameter; RA: right atrial; RV: right ventricular; LV: left ventricular; Antiplatelets: aspirin, clopidogrel, ticagrelor; Antihypertensives: angiotensin-converting enzyme inhibitor, angiotensin receptor blocker, calcium-channel blocker, β-receptor blocker; PCI: percutaneous transluminal coronary intervention; CABG: coronary artery bypass grafting.
