# Supplementary material for: Development and validation of a novel risk score to predict 5-year mortality in patients with acute myocardial infarction in China: a retrospective study
Source: PeerJ. 2022 Jan 4;10:e12652. doi: 10.7717/peerj.12652 (PMC8740514; doi:10.7717/peerj.12652)
Supplement: Supplemental Information 6 — Abbreviations: BMI, body mass index; STEMI, ST segment elevation myocardial infarction; MI, myocardial infarction; Door-to-Balloon time, Time from hospital arrival to first balloon inflation;SBP, systolic blood pressure; DBP, diastolic blood pressure; HR, heart rate; WBC, white blood cell; Hb, Hemoglobin; PLT, platelet; ALT, alanine transaminase; FBG, fast blood glucose; LDL-C, low-density lipoprotein cholesterol; NT-proBNP, N-terminal pro-brain natriuretic peptide; CK-MB, creatine kinase isoenzyme; cTnI, cardiac troponin I; LVEF, left ventricular ejection fraction; LA, left atrial; LVDd, left ventricular end-diastolic diameter; RA, right atrial; RV, right ventricular; LV, left ventricular; Antiplatelets, aspirin, clopidogrel, ticagrelor; Antihypertensives, angiotensin-converting enzyme inhibitor, angiotensin receptor blocker, calcium-channel blocker, β-receptor blocker; AF, atrial fibrillation; PCI, percutaneous transluminal coronary intervention; CABG, coronary artery bypass grafting. [file peerj-10-12652-s006.doc]

**Table S3 The Missing Data for Each Variable of AMI Patients in the Development and Validation Cohorts.**

| **Variables** | **Missing data (n, % of the development and validation cohorts)** | |
| --- | --- | --- |
|  | **Development Cohort (n=1471)** | **Validation Cohort (n=1251)** |
| Age, years | 0 (0) | 0 (0) |
| Gender (male), n (%) | 0 (0) | 0 (0) |
| BMI, kg/m^2^ | 42 (2.9) | 0 (0) |
| Current smoking, classifications | 0 (0) | 0 (0) |
| ST segment depression | 0 (0) | 3 (0.2) |
| STEMI, n (%) | 0 (0) | 3 (0.2) |
| Acute Anterior MI, n (%) | 0 (0) | 0 (0) |
| Left main coronary lesion, n (%) | 262 (17.8) | 0 (0) |
| Coronary multivessel lesion, n (%) | 262 (17.8) | 0 (0) |
| Door-to-Balloon time >4 hours, n (%) | 2 (0.1) | 0 (0) |
| Cardiac Arrest, n (%) | 0 (0) | 0 (0) |
| Killip, classifications | 0 (0) | 2 (0.2) |
| HR, beats/min | 0 (0) | 0 (0) |
| Systolic Blood Pressure | 43 (2.9) | 0 (0) |
| Diastolic Blood Pressure | 43 (2.9) | 0 (0) |
| WBC, *10^9^/L | 9 (0.6) | 0 (0) |
| Hb, g/L | 9 (0.6) | 0 (0) |
| PLT, *10^9^/L | 11 (0.7) | 0 (0) |
| ALT, U/L | 55 (3.7) | 0 (0) |
| Cr, μmol/L | 12 (0.8) | 0 (0) |
| FBG, mmol/L | 71 (4.8) | 0 (0) |
| LDL-C, mmol/L | 56 (3.8) | 0 (0) |
| NT-proBNP, pg/ml | 72 (4.9) | 103 (8.2) |
| cTnI, μg/L | 7 (0.5) | 0 (0) |
| Prior MI, n (%) | 0 (0) | 0 (0) |
| Prior PCI, n (%) | 0 (0) | 0 (0) |
| History of Hypertension, n (%) | 0 (0) | 0 (0) |
| History of Diabetes, n (%) | 0 (0) | 0 (0) |
| History of Stroke, n (%) | 0 (0) | 0 (0) |
| History of AF, n (%) | 0 (0) | 0 (0) |
| LVEF, % | 285 (19.4) | 191 (15.3) |
| LA, mm | 285 (19.4) | 191 (15.3) |
| LVDd, mm | 285 (19.4) | 191 (15.3) |
| RA, mm | 285 (19.4) | 191 (15.3) |
| RV, mm | 285 (19.4) | 191 (15.3) |
| Aortic Regurgitation, n (%) | 285 (19.4) | 191 (15.3) |
| Mitral Regurgitation, n (%) | 285 (19.4) | 191 (15.3) |
| Tricuspid Regurgitation, n (%) | 285 (19.4) | 191 (15.3) |
| Pulmonary Regurgitation, n (%) | 285 (19.4) | 191 (15.3) |
| Decreased Left Ventricular Compliance, n (%) | 285 (19.4) | 191 (15.3) |
| Antiplatelets Therapy, n (%) | 0 (0) | 0 (0) |
| Statins Therapy, n (%) | 0 (0) | 0 (0) |
| Antihypertensives Therapy, n (%) | 0 (0) | 0 (0) |
| PCI, n (%) | 2 (0.1) | 0 (0) |
| CABG, n (%) | 0 (0) | 0 (0) |
| Survival Time, months | 9 (0.6) | 65 (5.2) |
| Death, n (%) | 9 (0.6) | 65 (5.2) |

**Abbreviations:** BMI: body mass index; STEMI: ST segment elevation myocardial infarction; MI: myocardial infarction; Door-to-Balloon time: Time from hospital arrival to first balloon inflation; SBP: systolic blood pressure; DBP: diastolic blood pressure; HR: heart rate; WBC: white blood cell; Hb: Hemoglobin; PLT: platelet; ALT: alanine transaminase; FBG: fast blood glucose; LDL-C: low-density lipoprotein cholesterol; NT-proBNP: N-terminal pro-brain natriuretic peptide; CK-MB: creatine kinase isoenzyme; cTnI: cardiac troponin I; LVEF: left ventricular ejection fraction; LA: left atrial; LVDd: left ventricular end-diastolic diameter; RA: right atrial; RV: right ventricular; LV: left ventricular; Antiplatelets: aspirin, clopidogrel, ticagrelor; Antihypertensives: angiotensin-converting enzyme inhibitor, angiotensin receptor blocker, calcium-channel blocker, β-receptor blocker; AF: atrial fibrillation; PCI: percutaneous transluminal coronary intervention; CABG: coronary artery bypass grafting.
