# Supplementary material for: Development and validation of a novel risk score to predict 5-year mortality in patients with acute myocardial infarction in China: a retrospective study
Source: PeerJ. 2022 Jan 4;10:e12652. doi: 10.7717/peerj.12652 (PMC8740514; doi:10.7717/peerj.12652)
Supplement: Supplemental Information 7 — Abbreviations: LASSO, least absolute shrinkage and selection operator; PCI, percutaneous transluminal coronary intervention; Antihypertensives, angiotensin-converting enzyme inhibitor, angiotensin receptor blocker, calcium-channel blocker, β-receptor blocker; Door-to-Balloon time, Time from hospital arrival to first balloon inflation; FBG, fast blood glucose; RA, right atrial; LVDd, left ventricular end-diastolic diameter; Cr, creatinine; HR, heart rate; Hb, hemoglobin; LVEF, left ventricular ejection fraction; NT-proBNP, N-terminal pro-brain natriuretic peptide. [file peerj-10-12652-s007.doc]

**Table S4 Variables Selected by LASSO Regression.**

| **Variables** | **Coefficient (cm/s)** |
| --- | --- |
| Cardiac arrest (yes vs no) | 31.691 |
| Statins therapy (yes vs no) | -26.191 |
| PCI (yes vs no) | -13.266 |
| History of Stroke (yes vs no) | 5.235 |
| Killip, classifications | 5.216 |
| Decreased Left Ventricular Compliance (yes vs no) | -0.173 |
| Antihypertensive therapy, classifications | -0.420 |
| Door-to-Balloon time > 4h (yes vs no) | 0.690 |
| FBG, mmol/L | 0.358 |
| Mitral Regurgitation (yes vs no) | 0.563 |
| RA, mm | 0.232 |
| Age, y | 0.382 |
| LVDd, mm | 0.471 |
| Cr, μmol/L | 0.100 |
| HR, beats/min | 0.030 |
| Hb, g/L | -0.041 |
| LVEF, % | -0.025 |
| NT-proBNP, pg/ml | 0.002 |

**Abbreviations:** LASSO: least absolute shrinkage and selection operator; PCI: percutaneous transluminal coronary intervention; Antihypertensives: angiotensin-converting enzyme inhibitor, angiotensin receptor blocker, calcium-channel blocker, β-receptor blocker; Door-to-Balloon time: Time from hospital arrival to first balloon inflation; FBG: fast blood glucose; RA: right atrial; LVDd: left ventricular end-diastolic diameter; Cr: creatinine; HR: heart rate; Hb: hemoglobin; LVEF: left ventricular ejection fraction; NT-proBNP: N-terminal pro-brain natriuretic peptide.
