# Supplementary material for: Development and validation of a novel risk score to predict 5-year mortality in patients with acute myocardial infarction in China: a retrospective study
Source: PeerJ. 2022 Jan 4;10:e12652. doi: 10.7717/peerj.12652 (PMC8740514; doi:10.7717/peerj.12652)
Supplement: Supplemental Information 8 — * Dropped was not selected as a predictor in LASSO model Abbreviations: IncMSE, Increase in mean squared error; LASSO, least absolute shrinkage and selection operator; NT-proBNP, N-terminal pro-brain natriuretic peptide; PCI, percutaneous transluminal coronary intervention; Cr, creatinine; FBG, fast blood glucose; LVDd, left ventricular end-diastolic diameter; Hb, hemoglobin; LVEF, left ventricular ejection fraction; HR, heart rate; Door-to-Balloon time, Time from hospital arrival to first balloon inflation; RA, right atrial; HR, heart rate; WBC, white blood cell. [file peerj-10-12652-s008.doc]

**Table S5 Variables Selected by Random Forest Algorithm and LASSO Regression.**

| **Variables** | **Random Forest, % IncMSE** | **LASSO, Coefficient (cm/s)** |
| --- | --- | --- |
| NT-proBNP, pg/ml | 42.352 | 0.002 |
| Cardiac arrest (yes vs no) | 28.297 | 31.691 |
| Killip, classifications | 26.032 | 5.216 |
| PCI (yes vs no) | 25.168 | -13.266 |
| Age, y | 24.022 | 0.382 |
| Cr, μmol/L | 23.689 | 0.100 |
| History of Stroke (yes vs no) | 23.268 | 5.235 |
| Antihypertensive therapy, classifications | 23.011 | -0.420 |
| FBG, mmol/L | 22.651 | 0.358 |
| LVDd, mm | 22.136 | 0.471 |
| Hb, g/L | 22.013 | -0.041 |
| Statins therapy (yes vs no) | 21.731 | -26.191 |
| Mitral Regurgitation (yes vs no) | 21.658 | 0.563 |
| LVEF, % | 21.477 | -0.025 |
| HR, beats/min | 21.312 | 0.030 |
| Door to Balloon time >4h (yes vs no) | 21.172 | 0.690 |
| RA, mm | 20.852 | 0.232 |
| Decreased Left Ventricular Compliance (yes vs no) | 20.639 | -0.173 |
| WBC | 20.475 | Dropped |
| Admission Diastolic Blood Pressure | 20.009 | Dropped |

* Dropped was not selected as a predictor in LASSO model

**Abbreviations:** IncMSE: Increase in mean squared error; LASSO: least absolute shrinkage and selection operator; NT-proBNP: N-terminal pro-brain natriuretic peptide; PCI: percutaneous transluminal coronary intervention; Cr: creatinine; FBG: fast blood glucose; LVDd: left ventricular end-diastolic diameter; Hb: hemoglobin; LVEF: left ventricular ejection fraction; HR: heart rate; Door-to-Balloon time: Time from hospital arrival to first balloon inflation; RA: right atrial; HR: heart rate; WBC: white blood cell.
