# Supplementary material for: Development and validation of a novel risk score to predict 5-year mortality in patients with acute myocardial infarction in China: a retrospective study
Source: PeerJ. 2022 Jan 4;10:e12652. doi: 10.7717/peerj.12652 (PMC8740514; doi:10.7717/peerj.12652)
Supplement: Supplemental Information 9 — Abbreviations: AIC, akaike information criterion; Sum of Sq, sum of squares; RSS, root sum squares; LVEF, left ventricular ejection fraction; Hb, hemoglobin; HR, heart rate; Door-to-Balloon time, Time from hospital arrival to first balloon inflation; Antihypertensive therapy, angiotensin-converting enzyme inhibitor, angiotensin receptor blocker, calcium-channel blocker, β-receptor blocker; RA, right atrial; FBG, fast blood glucose; Cr, creatinine; LVDd, left ventricular end-diastolic diameter; NT-proBNP, N-terminal pro-brain natriuretic peptide; PCI, percutaneous transluminal coronary intervention. [file peerj-10-12652-s009.doc]

**Table S6 Variable Selection by Backward Stepwise Regression Model.**

| **Variables** | **AIC** | **Sum of Sq** | **RSS** | **Included in final model** |
| --- | --- | --- | --- | --- |
| LVEF, % | 3160.0 | 0.002 | 167.50 | Excluded |
| Decreased Left Ventricular Compliance (yes vs no) | 3159.7 | 0.045 | 167.55 | Excluded |
| Mitral Regurgitation (yes vs no) | -3162.8 | 0.081 | 167.64 | Excluded |
| Hb, g/L | -3161.8 | 0.196 | 167.76 | Excluded |
| HR, beats/min | -3161.5 | 0.228 | 167.79 | Excluded |
| Door to Balloon time > 4h (yes vs no) | -3161.4 | 0.237 | 167.80 | Excluded |
| RA, mm | -3160.1 | 0.385 | 167.95 | Excluded |
| Antihypertensive therapy (yes vs no) | -3159.8 | 0.422 | 167.98 | Excluded |
| FBG, mmol/L | -3158.7 | 0.554 | 168.12 | Included |
| Stroke (yes vs no) | -3156.0 | 0.859 | 168.42 | Included |
| LVDd, mm | -3150.9 | 1.450 | 169.01 | Included |
| Statin therapy (yes vs no) | -3148.1 | 1.763 | 169.32 | Included |
| Cr, μmol/L | -3147.9 | 1.786 | 169.35 | Included |
| Killip, classifications | -3146.2 | 1.992 | 169.55 | Included |
| NT-proBNP, pg/ml | -3142.9 | 2.360 | 169.92 | Included |
| Age, y | -3138.2 | 2.909 | 170.47 | Included |
| Cardiac arrest (yes vs no) | -3125.6 | 4.380 | 171.94 | Included |
| PCI (yes vs no) | -3106.6 | 6.609 | 174.17 | Included |
| Final 10 variable model | -3162.8 | - | 167.64 | - |

**Abbreviations:** AIC: akaike information criterion; Sum of Sq: sum of squares; RSS: root sum squares; LVEF: left ventricular ejection fraction; Hb: hemoglobin; HR: heart rate; Door-to-Balloon time: Time from hospital arrival to first balloon inflation; Antihypertensive therapy: angiotensin-converting enzyme inhibitor, angiotensin receptor blocker, calcium-channel blocker, β-receptor blocker; RA: right atrial; FBG: fast blood glucose; Cr: creatinine; LVDd: left ventricular end-diastolic diameter; NT-proBNP: N-terminal pro-brain natriuretic peptide; PCI: percutaneous transluminal coronary intervention.
