# Supplementary material for: Development and validation of a novel risk score to predict 5-year mortality in patients with acute myocardial infarction in China: a retrospective study
Source: PeerJ. 2022 Jan 4;10:e12652. doi: 10.7717/peerj.12652 (PMC8740514; doi:10.7717/peerj.12652)
Supplement: Supplemental Information 10 — Abbreviations: AMI, acute myocardial infarction; NT-proBNP, N-terminal pro-brain natriuretic peptide; FBG, fast blood glucose; LVDd, left ventricular end-diastolic diameter; PCI, percutaneous transluminal coronary intervention. [file peerj-10-12652-s010.doc]

**Table S7 The Nomogram Score for Each Variable in AMI Patients.**

| **Variables** | | **Risk Score** |
| --- | --- | --- |
| **1.** | **Creatinine, μmol/L** |  |
|  | ≤ 35 | 7 |
|  | 36-70 | 0 |
|  | 71-105 | 22 |
|  | 106-140 | 28 |
|  | 141-176 | 36 |
|  | ≥ 177 | 43 |
| **2.** | **Cardiac Arrest** |  |
|  | No | 0 |
|  | Yes | 87 |
| **3.** | **Age, years** |  |
|  | < 40 | 0 |
|  | 40-49 | 20 |
|  | 50-59 | 40 |
|  | 60-69 | 60 |
|  | 70-79 | 80 |
|  | 80-89 | 100 |
| **4.** | **NT-proBNP, pg/ml** |  |
|  | 0-300 | 0 |
|  | ≥ 300 | 36 |
| **5.** | **Statins Therapy** |  |
|  | No | 80 |
|  | Yes | 0 |
| **6.** | **Stroke** |  |
|  | No | 0 |
|  | Yes | 27 |
| **7.** | **FBG, mmol/L** |  |
|  | 0-10 | 0 |
|  | ≥ 10 | 26 |
| **8.** | **LVDd, mm** |  |
|  | 0-60 | 0 |
|  | ≥ 60 | 33 |
| **9.** | **PCI** |  |
|  | No | 55 |
|  | Yes | 0 |
| **10.** | **Killip, classifications** |  |
|  | I | 0 |
|  | II | 24 |
|  | III | 48 |
|  | IV | 72 |

**Abbreviations:** AMI: acute myocardial infarction; NT-proBNP: N-terminal pro-brain natriuretic peptide; FBG: fast blood glucose; LVDd: left ventricular end-diastolic diameter; PCI: percutaneous transluminal coronary intervention.
