# Supplementary material for: Development and validation of a novel risk score to predict 5-year mortality in patients with acute myocardial infarction in China: a retrospective study
Source: PeerJ. 2022 Jan 4;10:e12652. doi: 10.7717/peerj.12652 (PMC8740514; doi:10.7717/peerj.12652)
Supplement: Supplemental Information 11 — Notes: aPoint estimates and 95% CIs are shown for all values except Hosmer-Lemeshow calibration statistic, for which the point estimate. Abbreviations: AMI, acute myocardial infarction; CI, confidence intervals; GRACE, Global Registry of Acute Coronary Events; KAMIR, Korea Acute Myocardial Infarction Registry; CAMI, China Acute Myocardial Infarction. [file peerj-10-12652-s011.doc]

**Table S8 Comparison of C2ABS2-GLPK Score with GRACE, KAMIR and CAMI Scores for the Prediction of 5-Year Mortality in Patients with AMI.**

|  | **Cohorts** | **C2ABS2-GLPK** | **GRACE** | **KAMIR** | **CAMI** |
| --- | --- | --- | --- | --- | --- |
| c-statistic,  (95% CI) | Development | 0.811  (0.786-0.836) | 0.728 (0.697-0.759) | 0.783 (0.758-0.808) | 0.558 (0.523-0.593) |
|  | Validation | 0.787  (0.756-0.818) | 0.733  (0.698-0.768) | 0.769 (0.736-0.802) | 0.528 (0.485-0.571) |
| Hosmer-Lemeshow ^a^ | Development | 5.277 (0.728) | 6.780 (0.561) | 1.038 (0.998) | 2.717 (0.951) |
|  | Validation | 9.495 (0.302) | 8.497 (0.387) | 6.471 (0.595) | 10.140 (0.255) |

**Notes:** ^a^Point estimates and 95% CIs are shown for all values except Hosmer-Lemeshow calibration statistic, for which the point estimate.

**Abbreviations:** AMI: acute myocardial infarction; CI: confidence intervals; GRACE: Global Registry of Acute Coronary Events; KAMIR: Korea Acute Myocardial Infarction Registry; CAMI: China Acute Myocardial Infarction.
