# Supplementary material for: Development and validation of a novel risk score to predict 5-year mortality in patients with acute myocardial infarction in China: a retrospective study
Source: PeerJ. 2022 Jan 4;10:e12652. doi: 10.7717/peerj.12652 (PMC8740514; doi:10.7717/peerj.12652)
Supplement: Supplemental Information 12 — Notes: aCalibration slope, β coefficient from the Cox proportional hazards model with the linear predictor used as the sole independent variable. Abbreviations: SD, Standard Deviation. [file peerj-10-12652-s012.doc]

**Table S9 Internal and External Calibration of C2ABS2-GLPK Model Using 10×10 Cross-Validation Technique.**

| **Follow-up, years** | **Parameters, mean (SD)** | **Cohorts** | **C2ABS2-GLPK Score** |
| --- | --- | --- | --- |
| 1-year | Calibration Slope^a^ | Development | 0.992 (0.009) |
|  |  | Validation | 0.991 (0.001) |
|  | Intercept | Development | 0.416 (0.079) |
|  |  | Validation | 0.218 (0.051) |
| 2-year | Calibration Slope^a^ | Development | 0.989 (0.009) |
|  |  | Validation | 0.987 (0.001) |
|  | Intercept | Development | 0.512 (0.095) |
|  |  | Validation | 0.297 (0.059) |
| 3-year | Calibration Slope^a^ | Development | 0.986 (0.012) |
|  |  | Validation | 0.983 (0.010) |
|  | Intercept | Development | 0.584 (0.106) |
|  |  | Validation | 0.370 (0.071) |
| 4-year | Calibration Slope^a^ | Development | 0.984 (0.014) |
|  |  | Validation | 0.980 (0.012) |
|  | Intercept | Development | 0.642 (0.117) |
|  |  | Validation | 0.421 (0.076) |
| 5-year | Calibration Slope^a^ | Development | 0.988 (0.014) |
|  |  | Validation | 0.976 (0.012) |
|  | Intercept | Development | 0.716 (0.135) |
|  |  | Validation | 0.473 (0.082) |

**Notes:** ^a^Calibration slope: β coefficient from the Cox proportional hazards model with the linear predictor used as the sole independent variable.

**Abbreviations:** SD: Standard Deviation.
