# Supplementary material for: Development and validation of a novel risk score to predict 5-year mortality in patients with acute myocardial infarction in China: a retrospective study
Source: PeerJ. 2022 Jan 4;10:e12652. doi: 10.7717/peerj.12652 (PMC8740514; doi:10.7717/peerj.12652)
Supplement: Supplemental Information 13 — Abbreviations: CI, confidence intervals. [file peerj-10-12652-s013.doc]

**Table S10 Comparison of 5-Year Mortality among C2ABS2-GLPK Risk Strata in Development and Validation Cohorts.**

| **Risk**  **Groups** | **Development Cohort** | | **Validation Cohort** | |
| --- | --- | --- | --- | --- |
|  | **Prevalence**  **of death (%)** | **Hazard Ratio (95%CI)** | **Prevalence**  **of death (%)** | **Hazard Ratio (95%CI)** |
| Low (0-148) | 575 (6.6) | reference | 843 (8.8) | reference |
| Medium (149-218) | 363 (15.2) | 2.4 (1.6-3.6) | 277 (19.9) | 2.6 (1.8-3.6) |
| High (≥219) | 533 (37.3) | 7.2 (5.1-10.2) | 131 (43.5) | 6.5 (4.6-9.1) |

**Abbreviations:** CI: confidence intervals.

**Table S8 Sensitivity Analysis: Discrimination of the C2ABS2-GLPK Model with Complete Case Data**

| **C2ABS2-GLPK Model** | **Development Cohort** | | **Validation Cohort** | |
| --- | --- | --- | --- | --- |
|  | **No. of patients** | **C-Statistic,**  **95% CI** | **No. of patients** | 1. **Statistic,**   **95% CI** |
| **Imputed Data** | 1471 | 0.817 (0.792-0.842) | 1251 | 0.794 (0.763-0.825) |
| **Complete Case Data** | 1071 | 0.812(0.781-0.843) | 919 | 0.783 (0.746-0.820) |

**Abbreviations:** CI: confidence intervals.

**Table S9 Performance of C2ABS2-GLPK, GRACE and KAMIR Scores in the Subgroups in the Development and External Validation Cohort.**

|  | **Risk Scores** | **Development Cohort** | | | **External Validation Cohort** | | |
| --- | --- | --- | --- | --- | --- | --- | --- |
|  |  | **Subgroups** | **Prevalence**  **of death (%)** | **C-stastics,**  **(95% CI)** | **Subgroups** | **Prevalence**  **of death (%)** | **AUC,**  **(95% CI)** |
| Smoke | C2ABS2-GLPK | Yes  N=825 | 140 (16.97) | 0.828 (0.795-0.861 ) | Yes  N=710 | 86 (12.11) | 0.778 (0.727-0.829) |
|  | GRACE |  |  | 0.716 (0.669-0.763) |  |  | 0.724 (0.669-0.779) |
|  | KAMIR |  |  | 0.79 (0.753-0.827) |  |  | 0.757 (0.706-0.808) |
|  | CAMI |  |  | 0.58 (0.531-0.629) |  |  | 0.575 (0.514-0.636 ) |
|  | C2ABS2-GLPK | No  N=646 | 152 (23.53) | 0.807 (0.770-0.844) | No  N=541 | 100 (18.48) | 0.801(0.762-0.840 ) |
|  | GRACE |  |  | 0.73 (0.689-0.771) |  |  | 0.739 (0.690-0.788) |
|  | KAMIR |  |  | 0.767 (0.730-0.804) |  |  | 0.772 (0.729-0.815) |
|  | CAMI |  |  | 0.524 (0.475-0.573) |  |  | 0.51 (0.453-0.567 ) |
| STEMI | C2ABS2-GLPK | Yes  N=922 | 159 (17.25) | 0.814 (0.779-0.849 ) | Yes  N=921 | 132 (14.33) | 0.785 (0.742-0.828 ) |
|  | GRACE |  |  | 0.742 (0.701-0.783) |  |  | 0.718 (0.667-0.769) |
|  | KAMIR |  |  | 0.786 (0.751-0.821 ) |  |  | 0.749 (0.702-0.796 ) |
|  | CAMI |  |  | 0.512 (0.467-0.557) |  |  | 0.516 (0.459-0.573) |
|  | C2ABS2-GLPK | No  N=549 | 133 (24.23) | 0.817 (0.782-0.852) | No  N=330 | 54 (16.36) | 0.819 (0.760-0.878) |
|  | GRACE |  |  | 0.722 (0.675-0.769) |  |  | 0.741 (0.670-0.812) |
|  | KAMIR |  |  | 0.771 (0.732-0.810 ) |  |  | 0.79 (0.723-0.857 ) |
|  | CAMI |  |  | 0.538 (0.483-0.593) |  |  | 0.5 (0.406-0.594) |

**Abbreviations:** KAMIR: Korea Acute Myocardial Infarction Registry; GRACE: Global Registry of Acute Coronary Events; AUC: area under curve; CI: confidence intervals.

**Table S9 *(Continued).***

|  | **Risk Scores** | **Development Cohort** | | | **External Validation Cohort** | | |
| --- | --- | --- | --- | --- | --- | --- | --- |
|  |  | **Subgroups** | **Prevalence**  **of death (%)** | **AUC,**  **(95% CI)** | **Subgroups** | **Prevalence**  **of death (%)** | **AUC,**  **(95% CI)** |
| Type 2 Diabetes | C2ABS2-GLPK | Yes  N=478 | 104 (21.76) | 0.818 (0.777-0.859 ) | Yes  N=218 | 40 (18.35) | 0.824 (0.765-0.883) |
|  | GRACE |  |  | 0.723 (0.672-0.774) |  |  | 0.752 (0.672-0.832) |
|  | KAMIR |  |  | 0.793 (0.750-0.836) |  |  | 0.787 (0.718-0.856) |
|  | CAMI |  |  | 0.542 (0.483-0.601) |  |  | 0.573 (0.497-0.649) |
|  | C2ABS2-GLPK | No  N=993 | 188 (18.93) | 0.814 (0.783-0.845) | No  N=1033 | 146 (14.13) | 0.784 (0.749-0.819) |
|  | GRACE |  |  | 0.731 (0.692-0.770 ) |  |  | 0.725 (0.684-0.766) |
|  | KAMIR |  |  | 0.777 (0.746-0.808) |  |  | 0.76 (0.721-0.799) |
|  | CAMI |  |  | 0.565 (0.522-0.608 ) |  |  | 0.511 (0.462-0.560 ) |
| Gender | C2ABS2-GLPK | Female  N=421 | 105 (24.94) | 0.777 (0.730-0.824 ) | Female  N=340 | 73 (21.47) | 0.805 (0.758-0.852) |
|  | GRACE |  |  | 0.684 (0.631-0.737) |  |  | 0.765 (0.712-0.818) |
|  | KAMIR |  |  | 0.749 (0.704-0.794) |  |  | 0.782 (0.737-0.827) |
|  | CAMI |  |  | 0.5 (0.441-0.559) |  |  | 0.528 (0.463-0.593 ) |
|  | C2ABS2-GLPK | Male  N=1050 | 187 (17.81) | 0.836 (0.807-0.865 ) | Male  N=911 | 113 (12.40) | 0.776 (0.733-0.819) |
|  | GRACE |  |  | 0.74 (0.701-0.779) |  |  | 0.706 (0.657-0.755) |
|  | KAMIR |  |  | 0.792 (0.759-0.825) |  |  | 0.744 (0.697-0.791) |
|  | CAMI |  |  | 0.572 (0.529-0.615 ) |  |  | 0.543 (0.488-0.598) |

**Abbreviations:** KAMIR: Korea Acute Myocardial Infarction Registry; GRACE: Global Registry of Acute Coronary Events; AUC: area under curve; CI: confidence intervals.

*Table S9 (Continued).*

|  | **Risk Scores** | **Development Cohort** | | | **External Validation Cohort** | | |
| --- | --- | --- | --- | --- | --- | --- | --- |
|  |  | **Subgroups** | **Prevalence**  **of death (%)** | **AUC,**  **(95% CI)** | **Subgroups** | **Prevalence**  **of death (%)** | **AUC,**  **(95% CI)** |
| Obesity | C2ABS2-GLPK | BMI>24 Kg/m^2^  N=700 | 108 (15.43) | 0.833 (0.792-0.874) | BMI>24 Kg/m2  N=407 | 48 (11.79) | 0.815 (0.746-0.884) |
|  | GRACE |  |  | 0.744 (0.693-0.795) |  |  | 0.722 (0.648-0.796) |
|  | KAMIR |  |  | 0.797 (0.754-0.840 ) |  |  | 0.807 (0.746-0.868) |
|  | CAMI |  |  | 0.575 (0.518-0.632) |  |  | 0.558 (0.480-0.636) |
|  | C2ABS2-GLPK | BMI<24 kg/m^2^  N=771 | 184 (23.87) | 0.8 (0.769-0.831 ) | BMI<24 Kg/m^2^  N=844 | 138 (16.35) | 0.777 (0.742-0.812) |
|  | GRACE |  |  | 0.703 (0.662-0.744 ) |  |  | 0.729 (0.688-0.770) |
|  | KAMIR |  |  | 0.762 (0.729-0.795) |  |  | 0.748 (0.707-0.789) |
|  | CAMI |  |  | 0.548 (0.503-0.593) |  |  | 0.516 (0.467-0.565) |
| DL-C | C2ABS2-GLPK | LDL-C< 2.59 mmol/L  N=862 | 188 (21.81) | 0.817 (0.786-0.848) | LDL-C< 2.59 mmol/L  N=519 | 83 (15.99) | 0.777 (0.726-0.828) |
|  | GRACE |  |  | 0.742 (0.705-0.779) |  |  | 0.721 (0.664-0.778) |
|  | KAMIR |  |  | 0.776 (0.743-0.809) |  |  | 0.746 (0.693-0.799) |
|  | CAMI |  |  | 0.576 (0.531-0.621) |  |  | 0.548 (0.483-0.613) |
|  | C2ABS2-GLPK | LDL-C> 2.59 mmol/L  N=609 | 104 (17.08) | 0.814 (0.773-0.855) | LDL-C> 2.59 mmol/L  N=732 | 103 (14.07) | 0.812 (0.773-0.851) |
|  | GRACE |  |  | 0.698 (0.641-0.755) |  |  | 0.743 (0.696-0.790) |
|  | KAMIR |  |  | 0.792 (0.751-0.833) |  |  | 0.785 (0.742-0.828) |
|  | CAMI |  |  | 0.477 (0.422-0.532) |  |  | 0.489 (0.434-0.544 ) |

**Abbreviations:** KAMIR: Korea Acute Myocardial Infarction Registry; GRACE: Global Registry of Acute Coronary Events; AUC: area under curve; CI: confidence intervals.
