# Supplementary material for: Development and validation of a novel risk score to predict 5-year mortality in patients with acute myocardial infarction in China: a retrospective study
Source: PeerJ. 2022 Jan 4;10:e12652. doi: 10.7717/peerj.12652 (PMC8740514; doi:10.7717/peerj.12652)
Supplement: Supplemental Information 14 — Abbreviations: CI, confidence intervals. [file peerj-10-12652-s014.doc]

**Table S11 Sensitivity Analysis: Discrimination of the C2ABS2-GLPK Model with Complete Case Data.**

| **C2ABS2-GLPK Model** | **Development Cohort** | | **Validation Cohort** | |
| --- | --- | --- | --- | --- |
|  | **No. of patients** | **c-statistic, 95% CI** | **No. of patients** | **c-statistic, 95% CI** |
| **Imputed Data** | 1471 | 0.811 (0.786-0.836) | 1251 | 0.787 (0.756-0.818) |
| **Complete Case Data** | 1079 | 0.805 (0.774-0.836) | 971 | 0.780 (0.743-0.817) |

**Abbreviations:** CI: confidence intervals.
